# Variation between Hospitals with Regard to Diagnostic Practice, Coding Accuracy, and Case-Mix. A Retrospective Validation Study of Administrative Data versus Medical Records for Estimating 30-Day Mortality after Hip Fracture

**DOI:** 10.1371/journal.pone.0156075

**Published:** 2016-05-20

**Authors:** Jon Helgeland, Doris Tove Kristoffersen, Katrine Damgaard Skyrud, Anja Schou Lindman

**Affiliations:** 1 Quality Measurement Unit, Norwegian Institute of Public Health, Oslo, Norway; 2 Department of Registration, Institute of Population-Based Cancer Research, Cancer Registry of Norway, Oslo, Norway; Mayo Clinic, UNITED STATES

## Abstract

**Background:**

The purpose of this study was to assess the validity of patient administrative data (PAS) for calculating 30-day mortality after hip fracture as a quality indicator, by a retrospective study of medical records.

**Methods:**

We used PAS data from all Norwegian hospitals (2005–2009), merged with vital status from the National Registry, to calculate 30-day case-mix adjusted mortality for each hospital (n = 51). We used stratified sampling to establish a representative sample of both hospitals and cases. The hospitals were stratified according to high, low and medium mortality of which 4, 3, and 5 hospitals were sampled, respectively. Within hospitals, cases were sampled stratified according to year of admission, age, length of stay, and vital 30-day status (alive/dead). The final study sample included 1043 cases from 11 hospitals. Clinical information was abstracted from the medical records. Diagnostic and clinical information from the medical records and PAS were used to define definite and probable hip fracture. We used logistic regression analysis in order to estimate systematic between-hospital variation in unmeasured confounding. Finally, to study the consequences of unmeasured confounding for identifying mortality outlier hospitals, a sensitivity analysis was performed.

**Results:**

The estimated overall positive predictive value was 95.9% for definite and 99.7% for definite or probable hip fracture, with no statistically significant differences between hospitals. The standard deviation of the additional, systematic hospital bias in mortality estimates was 0.044 on the logistic scale. The effect of unmeasured confounding on outlier detection was small to moderate, noticeable only for large hospital volumes.

**Conclusions:**

This study showed that PAS data are adequate for identifying cases of hip fracture, and the effect of unmeasured case mix variation was small. In conclusion, PAS data are adequate for calculating 30-day mortality after hip-fracture as a quality indicator in Norway.

## Introduction

In recent years, public reporting of hospital quality indicators in general, and outcome indicators in particular, have been established in several health care systems and in international comparative studies [1–4]. In particular, mortality within a fixed time period after admission, for certain conditions, is in widespread use as a quality indicator.

The Norwegian Institute for Public Health reports one overall indicator (based on diagnosis groups leading to 80% of all deaths within 30 days) and three condition-specific indicators; i.e. first time acute myocardial infarction (AMI), stroke and hip fracture [5–8] [9]. Hospitals with case-mix adjusted mortality, defined as the probability of (all-cause) death within 30 days of hospitalization, that is significantly higher or lower compared to the (trimmed) hospital mean are identified for each indicator, and the outlier hospitals are reported. The results are published annually on the website of the Norwegian Directorate of Health, as part of the Norwegian Quality Indicator System, authorized by the Ministry of Health and Care Services.

Although 30-day mortality as a quality indicator is in wide-spread use, and has been found useful for quality improvement, see e.g. [10], the validity and usefulness of the indicators as measures of quality has been disputed, see e.g. [11],[12],[13],[14],[15],[16]. Studies have reported poor or variable coding quality [17],[18], as have administrative audits of coding practice in Norway [19, 20]. In our experience, many clinicians share the concerns about the validity of the indicators as meaningful measures of quality. In our view, the ultimate and important objective of quality indicators is to inform and support quality improvement efforts in hospitals, which requires that they have high credibility among practitioners and management. Two main objections against the mortality quality indicators based on PAS are:

1. the accuracy of the data, especially with regard to diagnostic coding
2. whether they capture the variation in case-mix between hospitals, or there are non-ignorable differences in patent frailty or case severity between hospitals

Still, patient administrative systems (PAS) are the least costly sources with complete coverage for routine reporting of quality indicators such as 30-day mortality, and often the only available. Hypothetically, the apparent mortality variation could be entirely due to misclassification or unobserved confounding. In the epidemiological literature, the need for assessment of unmeasured confounding for observational studies have been emphasized [21]. To the best of the authors’ knowledge, there are no published studies directly addressing these issues for the case of 30-day mortality used as a quality indicator. In particular, variability between hospitals in coding or patient risk has not been studied for the case of hip fracture. When using quality indicators, it is important to know the magnitude and effect of the major error sources such as statistical variation and biases. For detection and publication of outliers, this information must be quantified and presented in a form that can be incorporated and accounted for in the statistical model.

The purpose of this study was to assess the validity of PAS data for calculating 30-day mortality after hip fracture as a quality indicator, by performing a retrospective validation study of PAS data versus medical records addressing the following: 1) Are cases reliably identified and are the diagnostic criteria applied uniformly across hospitals? 2) Are there systematic between-hospital differences in patient risk, i.e. unmeasured confounders not accounted for by the use of PAS, and what is the magnitude of this systematic error? 3) What are the consequences of the potential systematic between-hospital differences observed, in particular the effect on outlier detection?

We report the findings from validating administrative data for patients with hip fracture as part of a larger study designed to answer the questions above for the three condition-specific indicators.

### Outline of paper

To begin with, we describe our sampling method. The sampling plan was designed to give high power to detect likely differences between hospitals. First, hospitals were divided into strata based on their 30-day mortality (calculated from PAS data). A stratified, random hospital sample was drawn. Within the selected hospitals, a random sample of hip fractures (according to PAS) was drawn, stratified after admission year and case severity. For the sample, medical records were abstracted and merged with PAS data.

The analysis falls in three main parts, aimed at the principal research objectives:

1. To establish a gold standard for diagnosis, an algorithm was devised to classify cases into four classes: definite or probable hip fractures, not hip fracture and cases where hip fracture could not be documented. The statistical analysis consisted in comparing positive predictive value (PPV) between hospitals.
2. To investigate case mix variation, data were analysed in three steps. Firstly, a joint PAS/clinical model for 30-day mortality was established. This model captured all available information. Secondly, the model was analysed to separate out the incremental information for predicting 30-day mortality contained in clinical, medical record data, compared to administrative (PAS) data alone. When calculating the routine, PAS-based estimates of 30-day mortality, this incremental information will appear as unobservable confounding. Thirdly, incremental information was analysed to find a measure of between-hospital variation.
3. Eventually, we looked at how this variation would appear as an error source when testing for hospital outliers.

## Methods

The study was approved by The Norwegian Ministry of Health and Care Services, The Data Inspectorate of Norway and The Regional Committee for Medical and Research Ethics. The study was based on data for a large number of individuals, collected after their hospital stay. Accordingly, obtaining written consent for a sufficiently complete sample would have been prohibitive, and approval was given to conduct the study without patient consent. No directly identifying information was recorded in the analysis data nor was otherwise available during the analysis.

The basis for this validation study of hip fracture cases are the PAS data used for the reporting of national 30-day mortality indicators in Norway. A brief description of data retrieval, data pre-processing and 30-day mortality estimation are given below, before the validation study is described in detail, including hospital and case selection, medical abstraction process and statistical methods. Estimation of 30-day mortality was a preliminary step used to obtain a representative hospital sample by stratification. In S1 Fig, we display the sampling method graphically.

### PAS data

PAS data from 2002–2009 were retrieved from all Norwegian hospitals providing acute care for hip fracture (n = 51), by an in-house software system developed for this purpose [6]. Each data record contained a unique record key and information from a single ward admission comprising admission category (i.e. elective or acute), diagnosis codes (both primary and secondary), codes for medical procedures, age, gender, as well as date and time of ward admission and discharge.

All permanent residents in Norway have a Personal Identification Number (PIN). The hospitals submitted PIN and the unique record key (and no medical information) to Statistics Norway. Statistics Norway prepared an encrypted PIN for all patients having a valid PIN and provided information from the National Registry: vital status (alive/dead/ emigrated) and date of death when applicable. We merged PAS data from hospitals and data from the National Registry, using the unique record key. Thus, linking of medical information from current and previous hospitalizations, date of death (in-or-out-of hospital) and tracking of patients between hospitals were possible. Ward admissions for each patient were linked into *episodes of care* when less than eight hours elapsed from time of discharge to the next ward admission. An episode of care included stays at different wards within one hospital and stays at other hospitals if the patient was transferred between hospitals. Admission category (elective/acute) was identified from the first ward admission in the episode of care. Each episode of care comprised diagnoses and procedure information from all ward stays within the same episode. One episode of care corresponded to one case in the analysis. Acute cases of hip fracture were identified according to ICD-10 codes S72.0–2, primary or secondary diagnosis, occurring at the first hospital if care at more than one hospital. Episodes following an initial hip fracture episode within 60 days were considered readmissions and excluded from the study population. Only patients aged 65 years and older were included.

For assessing the consequences of unmeasured clinical confounders for the validity of the routinely reported quality indicators, a supplementary administrative data set, covering data up to and including 2013, was retrieved from the Norwegian National Patient Registry.

### PAS-based estimation of 30 day mortality

Risk adjusted 30-day mortality for admissions in the period 2005–2009 was estimated by logistic regression. The following case-mix variables were included: age, gender, number of previous hospital admissions two years prior to actual admission, as well as the Charlson comorbidity index as revised in [22], and computed from the ICD-10 codes in [23]. The Charlson index was calculated from previous admissions three years prior to, but not including, the current episode of care. The Charlson index and the number of previous admissions were based on data from 2002–2009. Estimated hospital effects were compared to a reference value, defined as the 10% trimmed mean of the hospital effects (on the logistic scale) [7]. A detailed description of algorithms and methods can be found in [5]. Eventually, the hospitals were stratified according to 30-day mortality status (hereafter 30D status): low (L30D) if the hospital effect was significantly different from and at least *log*(1.2) ≈ 0.18 below the reference, high (H30D) mortality if significantly different from and at least *log*(1.2) above, and medium (M30D) for the remaining hospitals.

### Hospital and case sampling method for the validation study

The hospital and case-sampling method were optimized for the purpose of this study. All hospitals in the population, except one, agreed to participate. From the remaining 50, twelve hospitals were sampled with the groups L30D, M30D and H30D as strata. Sampling was subject to the following constraints: the three largest, regional hospitals had to be included, at most one hospital per hospital trust could be included, and a limit was set on the amount of imbalance between the four Norwegian hospital regions. Three L30D hospitals, four H30D hospitals and five M30D hospitals were included.

The initial total sample size of cases was chosen to be 2000, to achieve 90% test power for detecting a between hospital variation of PPV from 75% to 85%. From each hospital in the sample, 167 cases were drawn at random, stratified according to year of admission and severity (dead within 30 days of admission, alive with length of stay above median, alive with length of stay below median). For patients transferred between hospitals, we selected the records from the first hospital in the episode of care. Eventually, due to cost and time constraints, we excluded one hospital and the earliest years (2005–2006) from the sample, leaving eleven hospitals and admission from 2007–2009. The abstracter was instructed to start with the most recently admitted cases and continue in decreasing order of admission time for the duration of the abstraction time period allocated to the hospital in question. In total, the sample consisted of 1088 records.

### Medical record abstraction

Medical record abstraction was performed by a trained nurse. We developed a questionnaire specifically for the purpose of this study, in cooperation with clinical experts. The hip fracture questionnaire consisted of nine sections comprising 56 questions, which included free text, multiple choice and time/date questions. The questionnaire included patient identification: patient ID, year of birth and gender and arrival status (alive/dead). Thereafter, information about diagnostic criteria: clinical symptoms at arrival and preoperative diagnostic findings, as well as clinical confounder variables for mortality prediction: chronic comorbidities (including the categories of the Charlson index, as revised in [22]), the American Society of Anesthesiologists’ physical status (ASA) score, physiological parameters and laboratory variables. Finally, in case of death, information regarding time and cause of death was also registered. The items were designed for recording and coding of existing information, avoiding subjective judgment as far as possible.

Data were entered in a database using an in-house developed web application. The web application was installed on encrypted laptops, and the data saved in a database via a safe VPN-connection.

A total number of 50 cases from one hospital were selected to be independently abstracted by a second abstracter, using a reduced set of items from the questionnaire.

### Statistical methods

#### Case identification and diagnostic criteria

**Fig 1:**
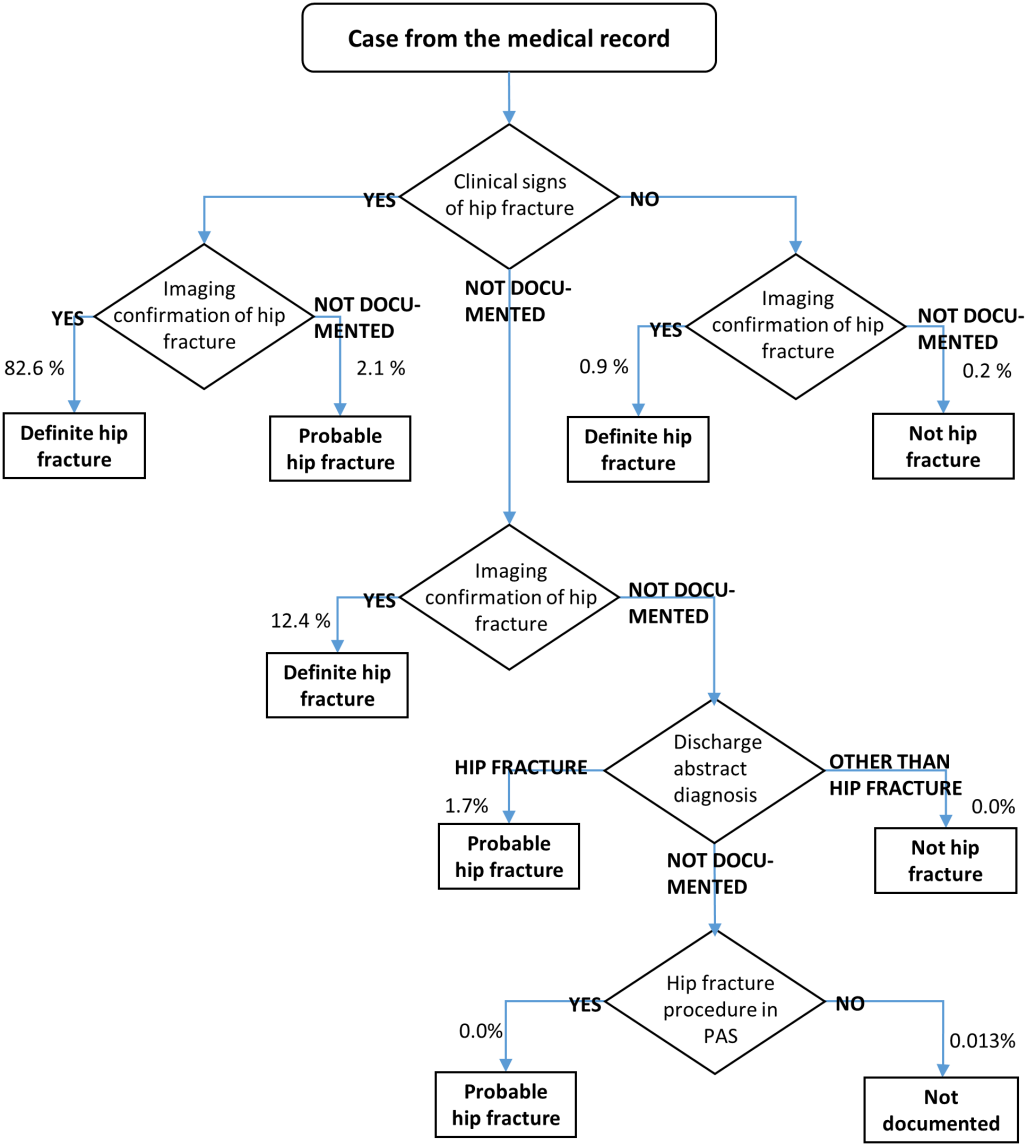
Algorithm for case classification. Number of cases at each step is also shown.

Data from the record abstraction were merged with the PAS data. Cases were initially classified as definite or probable hip fractures based on imaging confirmation and clinical signs, both taken from the medical records. Patient with legs that would not bear weight or were unnaturally rotated, were regarded as having clinical signs of hip fracture. Imaging confirmation was based on conventional X-ray, CT or MR. For some cases, neither imaging confirmation nor clinical signs could be documented. These cases were eventually resolved using administrative data on procedures (from PAS) and the diagnosis codes and verbal description, read from the discharge abstract of the records. These cases were classified as definite hip fracture, probable hip fracture, no hip fracture or “Not documented”, according to the algorithm in Fig 1.

#### Estimation of positive predictive value

To answer the first study objective, we estimated positive predictive value (PPV) and analysed whether there were differences in the hospitals’ PPVs for the hip fracture code. The stratified sampling plan, while increasing statistical precision in the analyses, precluded the use of standard statistical methods. Means and proportions in the hospital sample, in particular the proportions of definite and probable hip fractures, were thus estimated using the weights for stratified case sampling. For significance testing of differences between the hospitals’ positive predictive value (PPV), bootstrapping was used. The test statistic was the log likelihood ratio for homogeneity corresponding to Poisson distributed counts, applied to the cases not classified as hip fractures. With K independent Poisson counts *X*_1_,⋯,*X*_*K*_, this is given by

$$G=\sum_{X_{i}>0}X_{i}log\left(\frac{X_{i}}{\bar{X}}\right).$$ (1)

With hospital estimated proportions ${\hat{p}}_{1},\cdots ,{\hat{p}}_{K}$, the test statistic was modified to

$$G^{*}=\sum_{X^{*}{}_{i}>0}X_{i}{}^{*}log\left(\frac{X^{*}{}_{i}}{\bar{X^{*}}}\right),$$ (2)

where $\bar{X}$ denotes the mean of *X*_1_,⋯,*X*_*K*_.

Here, the counts have been replaced by predicted counts

$$X^{*}{}_{i}=M{\hat{p}}_{i},\;i=1,\cdots ,K,$$ (3)

where *M* is the median sample size per hospital and $\bar{X^{*}}$ denotes the mean of the predicted counts.

For the bootstrapping replications, the strata were assumed homogenous across hospitals under the null hypothesis. The reason for not using binomial likelihoods or *χ*^2^, was that the number and proportion of “Not documented” or non-hip fracture cases were zero or close to zero.

#### Confounding due to clinical variables

To answer the second aim; is there systematic between-hospital differences in patient risk, i.e. unmeasured confounders not accounted for by use of PAS, and what is the magnitude of this systematic error, a framework for sensitivity analysis as described in [24] was used. The above reference addresses the case of hypothetical confounding due to unobservable variables, whereas we have access to clinical data that enables us to obtain a measure of the confounding effects.

The case-mix confounder variables from medical records were summarized by three different risk scores: O-POSSUM (Orthopaedic Physiologic and Operative Severity Score for the enUmeration of Mortality and Morbidity) [25], NHFS (Nottingham hip fracture score) [26, 27] and SAPS II (Simplified Acute physiological Score II) [28]. We selected these scores according to the findings of systematic reviews [29, 30]. However, based on the available data, the variable set was not complete for the selected risk scores. Thus, we computed partial scores (see Table A in S2 Text for a tabulation of the variables used). The mean value was imputed in the records with missing values.

Assessment of the magnitude of case-mix variability was done in three stages: 1) Joint modelling of 30-day mortality given both PAS and clinical confounder variables from medical data. 2) Modelling the conditional distribution of clinical confounders, given PAS data. 3) Combining 1) and 2) in a model for 30-day mortality based on PAS data, but with clinical confounders regarded as unobservable.

Firstly, we fitted a retrospective logistic model for death within 30 days [31, 32] to the combined administrative and medical record data set. We used stepwise regression to select the risk score giving the best prediction. This model can be written as

$$log\left(\frac{p_{ij}}{1-p_{ij}}\right)=\mu +X_{ij}\beta +Z_{ij}\gamma ,\;\;\;\;\;j=1,\cdots ,N_{i},$$ (4)

where *p*_*ij*_ is the probability of death within 30 days of admission, *X*_*ij*_ is the vector of PAS covariates *Z*_*ij*_ is the vector of clinical risk scores for the j-the patient at the i-th hospital, and *N*_*i*_ is the total number of cases at hospital *i*, and *μ* is the constant term. Covariates were modelled by natural splines (age) (see e.g. [33], [34]) and fractional polynomials (Charlson index and number of previous admissions) [35].

Secondly, we estimated the hospital-specific distributions of the selected risk score, conditional on the administrative variables, assuming that the hospital effects were shifts in mean:

$$Z_{ij}=\;\nu +\;\alpha_{i}+X_{ij}\xi +\varepsilon_{ij}.$$ (5)

Here, *α*_*i*_ is the effect of hospital i and reflects the excess risk score at this hospital that cannot be explained by PAS covariates *X*_*ij*_. We regard the hospital effects *α*_*i*_ as random variables, sampled from the hospital population. The resulting linear mixed-effects model (LMM) was fit using restricted maximum likelihood (REML). We used unweighted REML, thus neglecting stratification. Exploratory analyses were done using ordinary least squares, including testing for non-zero hospital effects.

Thirdly, the estimated standard deviation of *α*_*i*_ was transformed to the linear predictor (logistic) scale of the logistic model for 30-day mortality, and regarded as a *measure of unobserved error in the logistic model based on administrative data*. The true model for outlier detection based on PAS data alone, incorporating the effect of unmeasured confounders, is given by

$$log\left(\frac{p_{ij}}{1-p_{ij}}\right)=\varphi +\theta_{i}+X_{ij}\beta +\zeta_{i}\;,\;\;\;\;\;\;\;j=1,\cdots ,m_{i},$$ (6)

where *p*_*ij*_ is the probability of death within 30 days of admission, *X*_*ij*_ is the vector of PAS covariates for the j-the patient at the *i*-th hospital, *m*_*i*_ the total number of cases at hospital *i*, and *φ* is a constant term. Here, the hospital effect *θ*_*i*_ is the apparent excess mortality on the linear predictor scale for hospital *i*, standardized by the requirement Σ_*i*_ *θ*_*i*_ = 0, and *ζ*_*i*_ is an unobservable, zero-mean term. In this model, *ν* and *X*_*ij*_*ξ* are absorbed into the constant and covariate terms.

#### Effect on of unmeasured confounding on outlier detection

The third aim of this study was to investigate the effect of unmeasured confounding on outlier detection, i.e. identification of hospitals with either significantly higher or lower mortality. We analysed the effect of the confounder term $\sigma_{\zeta }^{2}$ on hypothesis testing for non-zero hospital effects (i.e. outlier testing) based on administrative data. For outlier detection, the *θ*_*i*_ are estimated assuming in effect that *ζ*_*i*_ = 0, yielding the asymptotically normal test statistic $\theta_{i}{}^{*}$ with estimated variance $\sigma_{\theta }^{*2}$. The test statistic can be written

$$\theta_{i}{}^{*}={\hat{\theta }}_{i}+\zeta_{i},$$ (7)

where ${\hat{\theta }}_{i}$ is an (unobservable) estimate of the true hospital effect *θ*_*i*_. Assuming that $\sigma_{\zeta }^{2}$ is small, we approximate the true variance of the observable test statistic by $\sigma_{\theta }^{*2}+\sigma_{\zeta }^{2}$. For a given nominal test level and value of $\sigma_{\theta }^{*2}$, the true test level can be computed, assuming a normal distribution. $\sigma_{\theta }^{*2}$ depends largely on the case volume of hospital *i*, which is a more readily interpreted quantity. Accordingly, results have been expressed in terms of hospital case volume. To this end, an empirical relation was established by linear regression between case volume and $\sigma_{\theta }^{*2}$, based on the most recent Norwegian data [8].

All data pre-processing and statistical modelling was performed in R, versions 2.15.2 and 3.2.3. We used the function *lmer* from the package lme4 to estimate mixed linear models. To assess regression models, we used diagnostic plots and tested for highly influential observations using Cook’s distance. For logistic models, we also computed the Hosmer-Lemeshow C-statistic and the area under the ROC (Receiver Operating Characteristic).

## Results

### Sample

The initial case sample per hospital was 167 records. Data abstraction required about one hour per record on the average. Because of time and costs constraints, the number of cases was reduced (see Methods), and one (low-mortality) hospital was excluded. A total of 1088 journals were investigated. Of these, 21 cases were not retrieved from the journal systems. This gives an effective sample completeness of 98%. Furthermore, we excluded 24 questionnaires due to errors in registration: duplicated or wrong patient sample identification numbers and questionnaires with more than 24 hours difference in admission date and time. The final sample size was 1043 cases. The median number of cases per hospital was 97 with a range of 70–104. A table of patient characteristics is included in the supporting information (Table A in S2 Text).

#### Episode identification

For proper episode identification, the admission date and time from records should match the PAS date and time approximately, the fracture should have occurred before admission and the patient should not be dead on arrival. Since transferred patients were included in the sample only at the initial hospital, the sample should not include transfers. These conditions were satisfied in all but 24 (96%-98%) of cases (see Table A in S1 Text).

#### Diagnostic criteria and identification of cases

In Fig 1 we show the decision tree for identifying cases, classifying the cases as definite hip fracture, probable hip fracture, no hip fracture or “Not documented”. Table 1 shows the initial diagnosis classification from medical records, according to clinical signs and imaging evidence.

**Table 1:** Imaging and clinical diagnostic criteria.

	Clinical diagnostic criterion		
Imaging criterion	Clinical signs	No clinical signs	Not documented
**Imaging confirmation**	82.6	0.9	12.4
**Not documented**	2.1	0.2	1.8

Percentages, estimated using stratum weights. N = 1043. For patients not classified by clinical signs or imaging, diagnoses and procedure codes was investigated (see Fig 1).

Time of medical examination upon admission was recorded for all but 33 cases. Half of the cases were examined within 0.82 hours and 95% within 6.3 hours. The admission and main discharge diagnoses were recorded both as free text and ICD-10 codes in the medical records. The patient was regarded as having an admission diagnosis of hip fracture if this was indicated in the free text, or, if free text was missing, in the ICD-10 code. The remaining cases were classified as “Not hip fracture” or “Not documented”. Table 2 shows the admission and discharge diagnoses. In some cases, discharge diagnoses apparently referred to a department or hospital stay subsequent to the stay where hip fracture was initially treated.

**Table 2:** Admission and discharge diagnosis from records.

	Hip fracture	Other than hip fracture	Not documented
**Admission diagnosis**	88.7	7.6	3.7
**Discharge diagnosis**	96.6	0.9	2.5

Percentages, estimated using stratum weights. N = 1043.

Finally, the relevant procedure codes (as defined in Table B in S1 Text) were retrieved from the PAS data. Table 3 shows the distribution of procedure/no procedure as well as the discharge diagnoses across the four hip fracture categories. For five cases, final diagnosis was positively different from the imaging diagnosis.

**Table 3:** Final diagnosis from the classification tree versus presence of relevant procedures and discharge diagnosis.

	Relevant procedure		Discharge diagnosis		
Final diagnosis	No procedure	Procedure	Hip fracture	Other than hip fracture	Not documented
**Definite hip fracture**	9.8	86.1	92.8	0.7	2.4
**Probable hip fracture**	0.8	1.3	2.0	0.1	0.0
**No hip fracture**	0.2	0.1	0.2	0.0	0.0
**Not documented**	1.2	0.6	1.7	0.1	0.1

Percentages, estimated using stratum weights. N = 1043

#### Positive predictive value

As shown in Table 4, the overall PPV for definite hip fracture was 95.9%. This was not significantly associated with hospital (p = 0.14). The PPV for definite or probable hip fracture was 99.7%. This was not significantly associated with hospital (p = 0.22). The hospital-wise results can be found in Table C in S1 Text. These results thus show that there is no evidence of difference in PPV between hospitals.

**Table 4:** Final diagnosis.

Definite hip fracture	Probable hip fracture	Not hip fracture	Not documented
95.9	3.80	0.22	0.13

Percentages, estimated using stratum weights. N = 1043

#### Confounding due to clinical variables

Case mix characteristics: Fractures caused by cancer, presence of other significant trauma and other specified symptoms (dyspnea, nausea, abdominal pain, fever, confusion) or acute conditions (pneumonia, urine tract infection, sepsis, other infections, anemia, electrolyte disturbances, deep vein thrombosis, lung embolism) were recorded, as shown in Table D in S1 Text. The medical record-derived Charlson index [22] showed significant variation between hospitals, but did not appear associated with the 30D-status of the hospitals (results not shown). Comparing the Charlson index derived from medical records, with the PAS-derived index ([22, 23]) from previous admission within the last 3 years, we found that administrative data typically underestimate the Charlson index. The mean difference was 0.51, with the largest discrepancies for cancer and dementia. We provide further details in the supporting information (S3 Text). The proportion of missing values in the ASA score was high and varied widely between hospitals, with a mean of 47%, range 2%-100%. Accordingly, this variable could not be used in the analysis.

Clinical confounding: The first analysis step was to fit model (4) for the joint effect of administrative and clinical data on 30-day mortality (Table 5). The Hosmer-Lemeshow test for model fit had a p-value of 0.503, while the maximum Cook's distance was 0.045. The model showed fair predictive power, with an area under the ROC (Receiver Operating Characteristic) of 0.74. In the stepwise regression analysis, the partial O-POSSUM score was the only risk score retained in the final analysis. This variable was highly significant in the model (p = 0.0015).

**Table 5:** Joint PAS and medical record logistic model for 30-day mortality. N = 1043.

Variable	Estimate	Std. Error	z value	Pr(>|z|)
Natural spline in age, basis function 1	10.26	3.63	2.83	0.005
Natural spline in age, basis function 2	2.67	0.91	2.95	0.003
*Ch*(*Ch* + 1)^−1^ *log*(*Ch* + 1)	0.48	0.32	1.53	0.13
*Pre*(*Pre* + 1)	-0.28	0.52	-0.53	0.60
Female gender	0.30	0.35	0.86	0.39
Partial O-POSSUM score	0.12	0.04	3.18	0.0015

Ch, Charlson score derived from PAS data; Pre, number of previous admissions from the last two years

The next step was to fit model (5) to analyze the variation in the partial O-POSSUM score, conditional on the administrative variables. After preliminary data analysis, we used a square root transform for the score to ensure a relatively symmetric distribution without heavy outliers. Regression diagnostics showed no lack of fit, with a maximum Cook's distance of 0.034. A fixed effect test rejected the hypothesis of zero hospital effects *α*_*i*_ (p = 0.021). This shows that there exist systematic differences in patient risk between hospitals. Higher mortality 30D-status was also significantly associated with increasing partial O-POSSUM score (p = 0.030). The estimated standard deviation of the random hospital effect was 0.043.

Thirdly, the final estimate of the magnitude of the population hospital effect was determined, based on the preceding two models (4) and (5). Because of the square root transform, Taylor linearization was used. The effect of unmeasured confounders, on the linear predictor scale of the outlier detection model, had a standard deviation *σ*_*ζ*_ of 0.044. One of the hospitals had very strong influence on the between-hospital variation. The test for zero hospital effects was no longer significant after exclusion of this hospital (data not shown).

#### Effect of confounding variables on outlier detection

**Fig 2:**
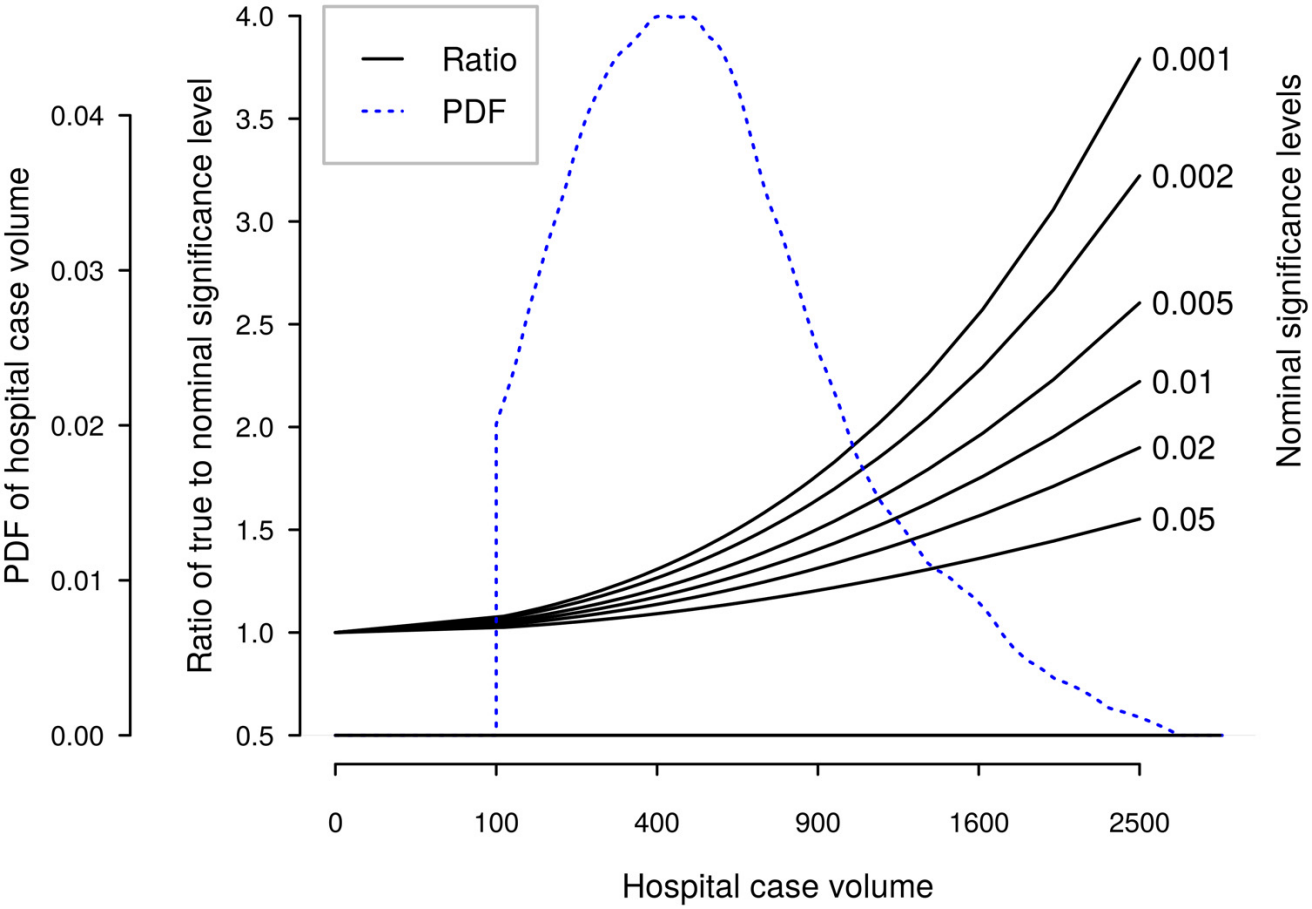
True test levels as function of number of cases, by nominal significance level. An estimate of probability density function (PDF) of Norwegian three-year hospital volumes is also shown. Note the nonlinear scale for hospital volume.

Using data from the most recent three-year period (2011–2013), we derived an empirical relation between standard deviation of the hospital effect estimate and hospital volume:

$$\sigma_{\theta }^{*}=3.74/\sqrt{N},$$

where $\sigma_{\theta }^{*}$ is the standard deviation of the estimate, and *N* is the number of cases. We used this relation to find the true significance level of tests for hospital outliers as a function of hospital case volume, for different nominal test levels. Note that *N* depends not only on yearly hospital volume, but also on the length of the observation period. Fig 2 shows the relative increase in true significance level for outlier tests, as a function of total hospital volume. For reference, we also display the most recent distribution of the Norwegian three-year hospital volumes.

## Discussion

We obtained a stratified sample of 1043 hip fracture cases from a sample of 11 Norwegian hospitals, and performed a medical record review. The PPV of a definite hip fracture episode identified in patient administrative data was 95.9%, increasing to 99.7% with probable cases included. We did not find any statistically significant differences in PPV between hospitals. We found a statistically significant, systematic between-hospital variation in 30-day mortality remaining after controlling for administrative data. However, the magnitude of this systematic variation was small to modest, but is a potential source of bias when using administrative data to identify hospitals that are mortality outliers. The consequences depend on the precise method used for outlier detection, but are unlikely to be important unless the hospital case volumes are very large. This finding is contrary to the warnings against using mortality statistics as quality indicators that have been frequently voiced in the literature. The Charlson comorbidity index [22, 23], based on administrative data from previous hospital admissions going three years back, tended to underestimate the index based on the records.

Our PPV results depend on the algorithm used to identify incident hip fracture cases from administrative data. First, episodes of subsequent admissions were constructed, possibly spanning more than one hospitals. The episode had to start with an emergency admission and hip fracture episodes from the previous 60 days were excluded. The choice of exclusion period has been studied previously, with the recommendation that this should not be less than 30 days [36].

Other investigators have studied coding accuracy and sensitivity for hip fracture. A similar study, based on administrative data from the Norwegian Patient Register (NPR) [37], used a sample stratified according to indication of hip fracture: 1) diagnosis and procedure codes, 2) only diagnosis codes and, 3) only procedure codes. Using their stratum-wise values for PPV, weighted with the relative proportions of these strata in our material, a PPV of 95.7% was obtained. Their completeness rate was lower than in our study. For the NOREPOS study [38], the same software was used for PAS data collection as in our study. In this study, a high concordance with data from local registries was reported; however, the case identification algorithm was somewhat different from ours. In a study from three Norwegian hospitals, the PPV of ICD-9 hip fracture codes was reported to be 84%, ranging from 82% to 85% over hospitals [17]. After exclusion of the categories “rehospitalisation for the same event” and “transferrals between hospitals”, the PPV increased to 95%. Due to our case identification algorithm, this PPV figure seems to be more appropriate for comparison. In a study of the Danish Arthroplasty Registry [39], the PPV for the diagnosis of fresh hip fracture was found to be poor, mainly due to incorrect identification of sequelae of older hip fractures.

Our results do not confirm the administrative audits’ general conclusions of poor coding quality in Norway [19, 20]. These studies did not report results specifically for hip fracture, and their objective was not to determine PPV. Studies of coding accuracy in other countries generally report high PPVs [40–42]. There are very few studies however, on variation in accuracy between hospitals (e.g. [43]), and none have used a representative nation-wide sample nor studied hip fractures, to our knowledge. Coding practices, in particular quality assurance of coding, differ between health systems. Our study extends previous results by investigating coding accuracy in a health care system where there is neither certification of coders nor regular, external audit of coding, and where ICD-10 coding is determined by resource use rather than cause of hospitalization. In general, our PPV values are in accordance with previous findings, although on the high side.

Two factors may have caused the relative insignificance of unobserved confounding: One is that age, number of previous admissions and Charlson index capture most of the patient risk variability. Another is that because in general, Norwegian patients are admitted to the nearest hospital for acute illness, and as the population also is relatively homogenous, patient variation tends to be averaged out, implying that any systematic variation in patient characteristics between hospitals is unlikely to be more then moderate.

We have not found any directly comparable studies of case-mix variation. Several studies have compared outlier detection models based on administrative and clinical data for surgery, generally reporting similar performance in terms of c-statistics [44–46]. The only study of orthopaedic surgery [47] found moderate correlation of risk scores based on the different data sets. Also, the same hospitals were identified as outliers. In a study of coronary bypass surgery, administrative data were found unsuitable due to imprecise identification of cases and outcomes, although the c-statistics were similar [48]. Coding of comorbidities or effects of clinical confounders have been studied in the case of acute myocardial infarction [49, 50]. These studies address sensitivity analysis directly and conclude that outlier detection based on administrative data is a valid approach. For stroke, however, contradictory results have been reported [51]. Our study extends previous results to hip fracture and another health system, as well as using a statistical model where the effect of unmeasured confounders is explicitly formulated and given a quantitative characterisation one can use directly to assess the validity of the quality indicator.

One strength of our study is the sampling method. The hospital sample was drawn at random from the complete national population, stratified on estimated 30-day mortality. The within-hospital stratification was optimized for the specific analysis objectives for this study, by oversampling of the most severe cases, thus yielding more precise estimates. The sampling frame was a complete, nation-wide set of patient administrative data. Another strength is that, as far as possible, only objective data were abstracted from records, leaving little room for the uncertainties of subjective interpretation and judgement. Also, our statistical model can be used for sensitivity analysis of outlier detection methods and other statistical procedures.

A consequence of the study design is the inability to estimate sensitivity. This would have required a prohibitively large sample from a very large set of hospital episodes. Note that when administrative data are used to study differences in mortality between hospitals, it is only the PPV (actually, differences in PPV between hospitals) that matters, not the sensitivity.

In our study, as in all retrospective studies, there remains the possibility that medical records are inaccurate or incomplete. Recording practices may differ between hospitals. We did not perform independent review of radiographs and other imaging data. We found indications that the retrieved information about comorbidities differed. However, efforts were made to abstract only information that was objective and presumably found in all hospitals. Even in prospective data registration, the variation between hospitals due to subjective judgement, different diagnostic practices etc. still remains. The double sampling sub study indicated (see S4 Text) that our record abstraction was reliable, though the sample size was too small for a definitive conclusion about error rates.

Also, the number of hospitals were limited by economic considerations. This is a cause of uncertainty in the estimates of the case-mix variability in the hospital population. However, the hospital sample was stratified according to mortality outlier status. Under the hypothesis of no true mortality variation, where outlier status is caused by bias alone, stratification should ensure a representative estimate of this bias. The moderate number of abstracted records places limitations on the precision of our analyses. The design yields good statistical power to detect variation that is roughly equally distributed among the hospital, but less power to detect a pattern where variation is small everywhere except for a few deviating hospitals.

The difficulty of finding information in the electronic record systems means that some variables could not be used in the analysis. The reasons are variation in what information is deemed necessary in the record systems as well as the largely unstructured organisation of these systems. It would have been possible to use other and more comprehensive data sources, such as operation theatre logs, curve systems and anaesthetists’ logs. In particular, due to incomplete recording in the electronic record systems, it was not possible to use the ASA score in our model, although it has been found to be a strong predictor of mortality after hip fracture [52]. The extra abstraction time required would then have to be weighed against the sample size that could be afforded. However, only systematic variation in record comprehensiveness would weaken the conclusions of this paper. In addition, data have been collected by only one abstracter. The work pattern and habits of the abstracter might have introduced bias in the data, particularly concerning retrieval of data items. Such bias would presumably apply equally to all hospitals.

In the statistical modelling, there is one distributional assumption that cannot be verified from our data alone. The estimation method (REML) for model (5) is derived on the assumption that the underlying hospital effects follow a normal distribution. However, the estimation of the hospital effects variance, which is the only parameter used subsequently, method has been shown to be reasonably robust with respect to deviation from normality [53, 54]. Without a much larger hospital sample, it is not reasonable to try to determine the hospital effects distribution with more precision.

Our derivation of a final diagnosis variable might be found arguable from an epidemiological point of view. However, we must stress that for the present purpose, only the between-hospitals consistency of the variable definition and abstraction is necessary. We regard the consistency as satisfactory.

In the case of unmeasured risk variation, one is naturally lead to considering whether the method of outlier detection could be modified to account for this source of error. Knowing the magnitude of the effect opens up for modified methods. However, this is outside the scope of the present paper.

### Conclusions

The main motivation for our study has been the need to validate 30-day mortality after hip fracture as a quality indicator for Norwegian hospitals, when based on administrative data. Other investigators may be concerned more with disease incidence or with the correctness of medical registries. We have demonstrated that the hip fracture code has a very high predictive value and shows little variation across hospitals. We have also found that the case-mix variation, not explained by the variables in administrative data, is relatively insignificant for quality indicators and outlier detection. Some consideration should be given, however, to the interpretation of quality indicators based on large hospital volumes, with 500–1000 or more cases. Relatively few hospitals seem to fall above this range in Norway and elsewhere, but this depends on the length of the time period used for reporting [8, 55–58]. When testing for mortality outliers, the problem of bias due to unmeasured confounders may be avoided by using an indifference interval in the hypotheses test, or by using a shorter time period (e.g. one year) for the very largest hospitals.

## Supporting Information

S1 Fig Preparation of sampling frame, sampling of hospitals and medical records. (TIF)

S1 Text Case classification. (PDF)

S2 Text Risk scores. (PDF)

S3 Text Comorbidity. (PDF)

S4 Text Double abstraction sub study. (PDF)

## References

[1] OECD. Health at a Glance 2013: OECD Publishing; 2013.

[2] Pearse RM, Moreno RP, Bauer P, Pelosi P, Metnitz P, Spies C, et al Mortality after surgery in Europe: a 7 day cohort study. Lancet. 2012;380(9847):1059–65. Epub 2012/09/25. 10.1016/s0140-6736(12)61148-9 ; PubMed Central PMCID: PMCPmc3493988. 22998715 PMC3493988

[3] Groene O, Kristensen S, Arah OA, Thompson CA, Bartels P, Sunol R, et al Feasibility of using administrative data to compare hospital performance in the EU. International journal for quality in health care: journal of the International Society for Quality in Health Care / ISQua. 2014;26 Suppl 1:108–15. Epub 2014/02/21. 10.1093/intqhc/mzu015 ; PubMed Central PMCID: PMCPmc4001688. 24554645 PMC4001688

[4] Gutacker N, Bloor K, Cookson R, Garcia-Armesto S, Bernal-Delgado E. Comparing hospital performance within and across countries: an illustrative study of coronary artery bypass graft surgery in England and Spain2015 2015-02-01 00:00:00. 28–34 p. 10.1093/eurpub/cku228 25690127

[5] Hassani S, Lindman AS, Kristoffersen DT, Tomic O, Helgeland J. 30-Day Survival Probabilities as a Quality Indicator for Norwegian Hospitals: Data Management and Analysis. PLoS One. 2015;10(9):e0136547 Epub 2015/09/10. 10.1371/journal.pone.0136547 . 26352600 PMC4564217

[6] Clench-Aas J, Helgeland J, Dimoski T, Gulbrendsen P, Hofoss D, Holmboe O, et al Methodological development and evaluation of 30-day mortality as quality indicator for Norwegian hospitals. Oslo: Norwegian Knowledge Centre for the Health Services, 2005. 29319970

[7] Helgeland J, Kristoffersen DT, Hassani S, Lindman AS, Dimoski T, Rygh LH. [30 day survival after admission to Norwegian hospitals in 2010 and 2011]. Oslo: Norwegian Knowledge Centre for the Health Services, 2013.

[8] Lindman AS, Hassani S, Kristoffersen DT, Tomic O, Dimoski T, Helgeland J. [30 day survival after admission to Norwegian hospitals for 2013]. Oslo: Norwegian Knowledge Centre for the health Services, 2014.

[9] Kristoffersen DT, Helgeland J, Clench-Aas J, Laake P, Veierod MB. Comparing hospital mortality—how to count does matter for patients hospitalized for acute myocardial infarction (AMI), stroke and hip fracture. BMC health services research. 2012;12:364 10.1186/1472-6963-12-364 23088745 PMC3526398

[10] Kristoffersen DT, Helgeland J, Waage HP, Thalamus J, Clemens D, Lindman AS, et al Survival curves to support quality improvement in hospitals with excess 30-day mortality after acute myocardial infarction, cerebral stroke and hip fracture: a before–after study. BMJ Open. 2015;5(3). 10.1136/bmjopen-2014-006741 PMC4386226 25808167

[11] Haug C. [Research without filter]. Tidsskrift for den Norske laegeforening: tidsskrift for praktisk medicin, ny raekke. 2005;125(23):3243 Epub 2005/12/06. . 16327842

[12] Lilford R, Pronovost P. Using hospital mortality rates to judge hospital performance: a bad idea that just won't go away. Bmj. 2010;340:c2016 10.1136/bmj.c2016 . 20406861

[13] McKee M. Hospital standardised mortality rates should not be used to make interhospital comparisons. Bmj. 2013;347:f6155 10.1136/bmj.f6155 . 24129379

[14] van Gestel YR, Lemmens VE, Lingsma HF, de Hingh IH, Rutten HJ, Coebergh JW. The hospital standardized mortality ratio fallacy: a narrative review. Medical care. 2012;50(8):662–7. 10.1097/MLR.0b013e31824ebd9f . 22410410

[15] Coombes R. Experts disagree about usefulness of hospital mortality data. Bmj. 2014;349:g5658 10.1136/bmj.g5658 . 25223438

[16] Ranstam J, Wagner P, Robertsson O, Lidgren L. Health-care quality registers: outcome-orientated ranking of hospitals is unreliable. The Journal of bone and joint surgery British volume. 2008;90(12):1558–61. 10.1302/0301-620X.90B12.21172 . 19043124

[17] Lofthus CM, Cappelen I, Osnes EK, Falch JA, Kristiansen IS, Medhus AW, et al Local and national electronic databases in Norway demonstrate a varying degree of validity. Journal of clinical epidemiology. 2005;58(3):280–5. 10.1016/j.jclinepi.2004.07.003 . 15718117

[18] van den Bosch WF, Silberbusch J, Roozendaal KJ, Wagner C. [Variations in patient data coding affect hospital standardized mortality ratio (HSMR)]. Nederlands tijdschrift voor geneeskunde. 2010;154:A1189 Epub 2010/02/25. . 20178667

[19] Jørgenvåg R, Hope ØB. [Quality of medical coding and ISF-reimbursement]. Oslo/Trondheim: SINTEF, 2005.

[20] Hoddevik GH. [Diagnosis versus code]. Tidsskrift for den Norske laegeforening: tidsskrift for praktisk medicin, ny raekke. 2005;125(21):2973–4. . 16276384

[21] Groenwold RH, Hak E, Hoes AW. Quantitative assessment of unobserved confounding is mandatory in nonrandomized intervention studies. Journal of clinical epidemiology. 2009;62(1):22–8. 10.1016/j.jclinepi.2008.02.011 . 18619797

[22] Quan H, Li B, Couris CM, Fushimi K, Graham P, Hider P, et al Updating and validating the Charlson comorbidity index and score for risk adjustment in hospital discharge abstracts using data from 6 countries. American journal of epidemiology. 2011;173(6):676–82. 10.1093/aje/kwq433 . 21330339

[23] Quan H, Sundararajan V, Halfon P, Fong A, Burnand B, Luthi JC, et al Coding algorithms for defining comorbidities in ICD-9-CM and ICD-10 administrative data. Medical care. 2005;43(11):1130–9. . 16224307 10.1097/01.mlr.0000182534.19832.83

[24] Lin DY, Psaty BM, Kronmal RA. Assessing the sensitivity of regression results to unmeasured confounders in observational studies. Biometrics. 1998;54(3):948–63. WOS:000080096700014. 9750244

[25] Mohamed K, Copeland GP, Boot DA, Casserley HC, Shackleford IM, Sherry PG, et al An assessment of the POSSUM system in orthopaedic surgery. The Journal of bone and joint surgery British volume. 2002;84(5):735–9. . 12188495 10.1302/0301-620x.84b5.12626

[26] Maxwell MJ, Moran CG, Moppett IK. Development and validation of a preoperative scoring system to predict 30 day mortality in patients undergoing hip fracture surgery. British journal of anaesthesia. 2008;101(4):511–7. 10.1093/bja/aen236 . 18723517

[27] Moppett IK, Parker M, Griffiths R, Bowers T, White SM, Moran CG. Nottingham Hip Fracture Score: longitudinal and multi-assessment. British journal of anaesthesia. 2012;109(4):546–50. 10.1093/bja/aes187 . 22728204

[28] Legall JR, Lemeshow S, Saulnier F. A New Simplified Acute Physiology Score (Saps-II) Based on a European North-American Multicenter Study. Jama-Journal of the American Medical Association. 1993;270(24):2957–63. WOS:A1993MM11900032. 10.1001/jama.270.24.2957 8254858

[29] Hu F, Jiang C, Shen J, Tang P, Wang Y. Preoperative predictors for mortality following hip fracture surgery: a systematic review and meta-analysis. Injury. 2012;43(6):676–85. 10.1016/j.injury.2011.05.017 . 21683355

[30] Strand K, Flaatten H. Severity scoring in the ICU: a review. Acta anaesthesiologica Scandinavica. 2008;52(4):467–78. 10.1111/j.1399-6576.2008.01586.x . 18339152

[31] Prentice R. Use of the logistic model in retrospective studies. Biometrics. 1976;32(3):599–606. . 963173

[32] Prentice RL, Pyke R. Logistic Disease Incidence Models and Case-Control Studies. Biometrika. 1979;66(3):403–11. 10.1093/biomet/66.3.403 WOS:A1979HX77100001.

[33] Wegman EJ, Wright IW. Splines in Statistics. Journal of the American Statistical Association. 1983;78(382):351–65. 10.2307/2288640 12280322

[34] Hastie T. Generalized additive models In: Chambers J, Hastie T, editors. Statistical Models in S: Chapman and Hall/CRC; 1991.

[35] Royston P, Altman DG. Regression Using Fractional Polynomials of Continuous Covariates—Parsimonious Parametric Modeling. Appl Stat-J Roy St C. 1994;43(3):429–67. WOS:A1994NV33700001.

[36] Vu T, Davie G, Barson D, Day L, Finch CF. Accuracy of evidence-based criteria for identifying an incident hip fracture in the absence of the date of injury: a retrospective database study. BMJ Open. 2013;3(7). 10.1136/bmjopen-2013-003222 23869105 PMC3717473

[37] Hoiberg MP, Gram J, Hermann P, Brixen K, Haugeberg G. The incidence of hip fractures in Norway -accuracy of the national Norwegian patient registry. BMC Musculoskelet Disord. 2014;15:372 10.1186/1471-2474-15-372 25394865 PMC4247646

[38] Omsland TK, Holvik K, Meyer HE, Center JR, Emaus N, Tell GS, et al Hip fractures in Norway 1999–2008: time trends in total incidence and second hip fracture rates: a NOREPOS study. European journal of epidemiology. 2012;27(10):807–14. 10.1007/s10654-012-9711-9 . 22870851

[39] Pedersen A, Johnsen S, Overgaard S, Soballe K, Sorensen HT, Lucht U. Registration in the danish hip arthroplasty registry: completeness of total hip arthroplasties and positive predictive value of registered diagnosis and postoperative complications. Acta orthopaedica Scandinavica. 2004;75(4):434–41. Epub 2004/09/17. . 15370588 10.1080/00016470410001213-1

[40] Hudson M, Avina-Zubieta A, Lacaille D, Bernatsky S, Lix L, Jean S. The validity of administrative data to identify hip fractures is high—a systematic review. Journal of clinical epidemiology. 2013;66(3):278–85. 10.1016/j.jclinepi.2012.10.004 . 23347851

[41] Ludvigsson JF, Andersson E, Ekbom A, Feychting M, Kim JL, Reuterwall C, et al External review and validation of the Swedish national inpatient register. Bmc Public Health. 2011;11 10.1186/1471-2458-11-450 WOS:000293023200001. PMC3142234 21658213

[42] Burns EM, Rigby E, Mamidanna R, Bottle A, Aylin P, Ziprin P, et al Systematic review of discharge coding accuracy. Journal of public health. 2012;34(1):138–48. 10.1093/pubmed/fdr054 21795302 PMC3285117

[43] Rosamond WD, Chambless LE, Sorlie PD, Bell EM, Weitzman S, Smith JC, et al Trends in the sensitivity, positive predictive value, false-positive rate, and comparability ratio of hospital discharge diagnosis codes for acute myocardial infarction in four US communities, 1987–2000. American journal of epidemiology. 2004;160(12):1137–46. WOS:000225663600001. 15583364 10.1093/aje/kwh341

[44] Parker JP, Li Z, Damberg CL, Danielsen B, Carlisle DM. Administrative versus clinical data for coronary artery bypass graft surgery report cards: the view from California. Medical care. 2006;44(7):687–95. Epub 2006/06/27. 10.1097/01.mlr.0000215815.70506.b6 . 16799364

[45] Aylin P, Bottle A, Majeed A. Use of administrative data or clinical databases as predictors of risk of death in hospital: comparison of models. Bmj. 2007;334(7602):1044 Epub 2007/04/25. 10.1136/bmj.39168.496366.55 ; PubMed Central PMCID: PMCPmc1871739. 17452389 PMC1871739

[46] Jang WM, Park J-H, Park J-H, Oh JH, Kim Y. Improving the Performance of Risk-adjusted Mortality Modeling for Colorectal Cancer Surgery by Combining Claims Data and Clinical Data. Journal of Preventive Medicine and Public Health. 2013;46(2):74–81. 10.3961/jpmph.2013.46.2.74 PMC3615382. 23573371 PMC3615382

[47] Gordon HSMD, Johnson MLP, Wray NPMDMPH, Petersen NJP, Henderson WGP, Khuri SFMD, et al Mortality After Noncardiac Surgery: Prediction From Administrative Versus Clinical Data. Medical care. 2005;43(2):159–67. 15655429 10.1097/00005650-200502000-00009

[48] Shahian DM, Silverstein T, Lovett AF, Wolf RE, Normand SL. Comparison of clinical and administrative data sources for hospital coronary artery bypass graft surgery report cards. Circulation. 2007;115(12):1518–27. Epub 2007/03/14. 10.1161/circulationaha.106.633008 . 17353447

[49] Austin PC, Tu JV, Alter DA, Naylor CD. The impact of under coding of cardiac severity and comorbid diseases on the accuracy of hospital report cards. Medical care. 2005;43(8):801–9. WOS:000230798100008. 16034294 10.1097/01.mlr.0000170414.55821.27

[50] Austin PC. The impact of unmeasured clinical variables on the accuracy of hospital report cards: A Monte Carlo study. Medical Decision Making. 2006;26(5):447–66. 10.1177/0272989x06290498 WOS:000240896700004. 16997924

[51] Fonarow GC, Pan W, Saver JL, Smith EE, Reeves MJ, Broderick JP, et al Comparison of 30-day mortality models for profiling hospital performance in acute ischemic stroke with vs without adjustment for stroke severity. Jama. 2012;308(3):257–64. 10.1001/jama.2012.7870 . 22797643

[52] Bjorgul K, Novicoff WM, Saleh KJ. American Society of Anesthesiologist Physical Status score may be used as a comorbidity index in hip fracture surgery. J Arthroplasty. 2010;25(6 Suppl):134–7. 10.1016/j.arth.2010.04.010 . 20537857

[53] Grilli L, Rampichini C. Specification of random effects in multilevel models: a review. Qual Quant. 2015;49(3):967–76. WOS:000353208800009.

[54] Verbeke G, Lesaffre E. The effect of misspecifying the random-effects distribution in linear mixed models for longitudinal data. Comput Stat Data An. 1997;23(4):541–56. WOS:A1997WG65100008.

[55] Kristensen PK, Thillemann TM, Johnsen SP. Is bigger always better? A nationwide study of hip fracture unit volume, 30-day mortality, quality of in-hospital care, and length of hospital stay. Medical care. 2014;52(12):1023–9. 10.1097/MLR.0000000000000234 . 25226544

[56] Browne JA, Pietrobon R, Olson SA. Hip fracture outcomes: does surgeon or hospital volume really matter? The Journal of trauma. 2009;66(3):809–14. 10.1097/TA.0b013e31816166bb . 19276758

[57] Hentschker C, Mennicken R. The Volume-Outcome Relationship and Minimum Volume Standards—Empirical Evidence for Germany. Health economics. 2014 10.1002/hec.3051 . 24700615

[58] Sund R. Modeling the volume-effectiveness relationship in the case of hip fracture treatment in Finland. BMC health services research. 2010;10:238 10.1186/1472-6963-10-238 20707899 PMC2931498

