# Supplementary figures and images for: Variation between Hospitals with Regard to Diagnostic Practice, Coding Accuracy, and Case-Mix. A Retrospective Validation Study of Administrative Data versus Medical Records for Estimating 30-Day Mortality after Hip Fracture
Source: PLoS One. 2016 May 20;11(5):e0156075. doi: 10.1371/journal.pone.0156075 (PMC4874695; doi:10.1371/journal.pone.0156075)

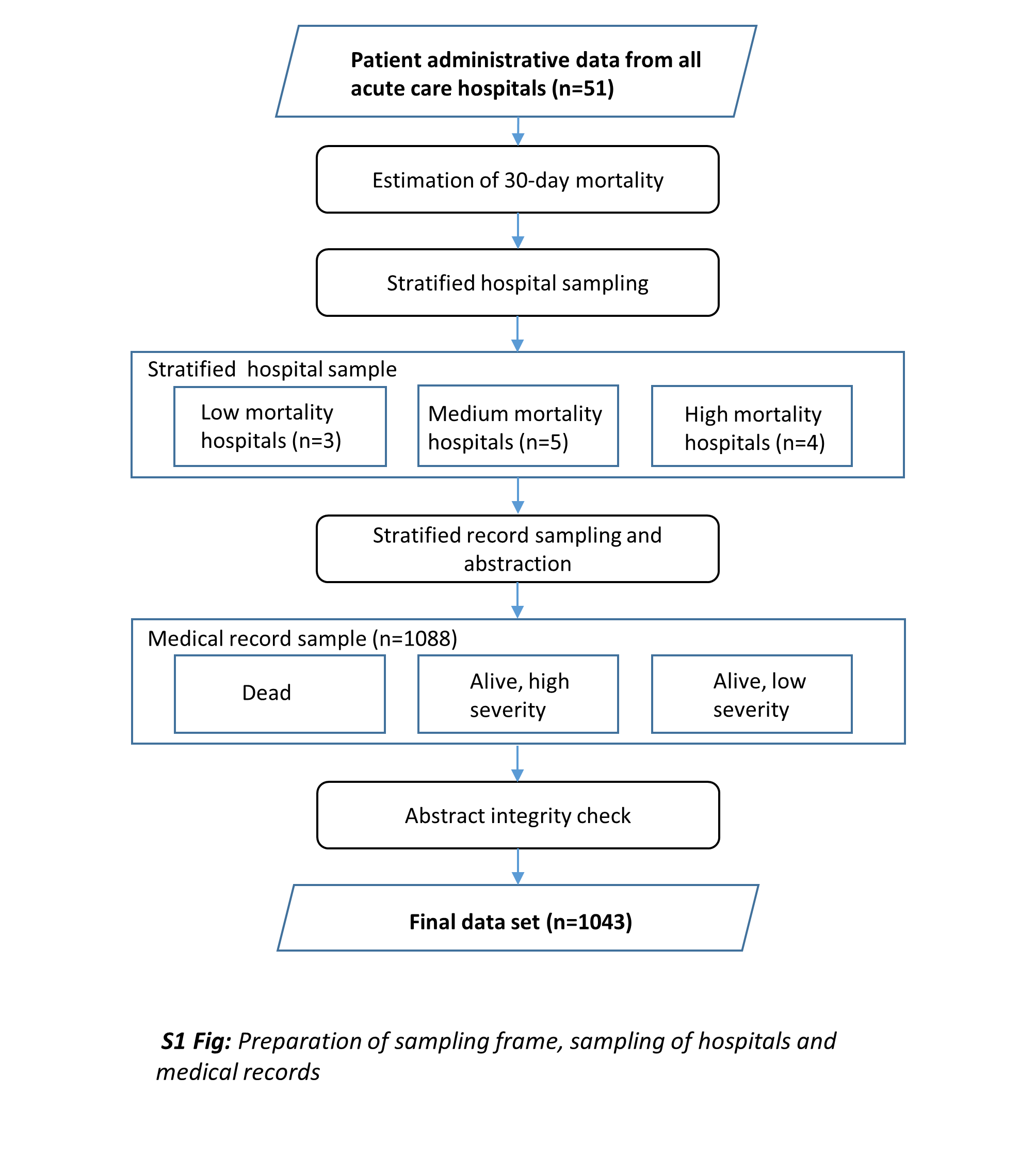

Supplement: S1 Fig — (TIF) [file pone.0156075.s001.tif]
