# Supplementary material for: Variation between Hospitals with Regard to Diagnostic Practice, Coding Accuracy, and Case-Mix. A Retrospective Validation Study of Administrative Data versus Medical Records for Estimating 30-Day Mortality after Hip Fracture
Source: PLoS One. 2016 May 20;11(5):e0156075. doi: 10.1371/journal.pone.0156075 (PMC4874695; doi:10.1371/journal.pone.0156075)
Supplement: S1 Text — (PDF) [file pone.0156075.s002.pdf]

S1 Text. Case classification

Table A*. *Episode identification. *Percentages, estimated using stratum weights. N=1043*

	Yes	No	Not documented
**Admission time difference < one hour**	95.7	3.6	0.7
**Admission time differences < eight hours**	98.1	1.2	0.7
**Alive one hour after admission**	98.4	0.0	1.6
**Admitted from home or long term care institution**	97.2	1.4	1.5
**Did the hip fracture occur before admission**	96.3	0.6	3.2

Procedure codes in the PAS data are from the Norwegian version of Nordic Medical Statistic Committee (NOMESCO) Surgical Procedure (NCSP) codes [1]. Table 2 shows procedure codes used in the identification of hip fractures.

Table B. NCSP codes for hip fracture operations

	**Type of fracture**		
**Procedure**	**Neck of femur**	**Pertrochanteric**	**Subtrochanteric**
**Internal fixation using screws, wire, rod, cerclage, pin, bioimplant, other or combined methods**	NFJ30, NFJ40, NFJ70, NFJ80		
**Partial prosthetic replacement, uncemented**	NFB01, NFB02, NFB09		
**Partial prosthetic replacement, cemented**	NFB11, NFB12, NFB19		
**Internal fixation using plate and/or screws**		NFJ61, NFJ81	NFJ62, NFJ82
**Internal fixation using intramedullary nail**		NFJ51	NFJ52

Table C. Final diagnosis classification, by hospital. *Percentages, estimated using stratum weights*

Hospital	Diagnosis			
	Definite hip fracture	Probable hip fracture	Not hip fracture	Not documented
Ringerike	93.0	6.2	0.0	1.0
Haukeland	95.0	4.7	0.0	0.0
StOlav	98.0	1.9	0.0	0.0
Tromsø	95.0	5.0	0.0	0.0
Kongsvinger	100	0.0	0.0	0.0
Arendal	95.0	3.1	1.0	1.0
Ahus	96.0	2.7	0.92	0.0
Vesterålen	99.0	0.98	0.0	0.0
Haraldsplass	97.0	3.1	0.0	0.0
Kristiansund	93.0	6.5	0.0	0.0
Fredrikstad	94.0	5.7	0.0	0.0

Table D. Percentages of fractured caused by cancer, presence of other significant trauma, other symptoms or acute conditions

	Yes	No/Not documented
Fractures caused by cancer	0.40	99.6
Presence of other significant trauma	4.50	95.5
Presence of other symptoms	4.20	95.8
Presence of acute conditions	0.28	99.7

Estimated using stratum weights. N=1043

References

1. KITH AS. [NCMP and NCSP - Clinical procedure codes 2009]. 2. edition ed. Oslo: Norwegian Directorate of Health; 2009.
