# Supplementary material for: Variation between Hospitals with Regard to Diagnostic Practice, Coding Accuracy, and Case-Mix. A Retrospective Validation Study of Administrative Data versus Medical Records for Estimating 30-Day Mortality after Hip Fracture
Source: PLoS One. 2016 May 20;11(5):e0156075. doi: 10.1371/journal.pone.0156075 (PMC4874695; doi:10.1371/journal.pone.0156075)
Supplement: S2 Text — (PDF) [file pone.0156075.s003.pdf]

# S2 Text. Risk scores

***Table A. Variables used for partial risk scores.*** *Descriptive statistics, estimated using stratum weights. N=1043*

| Variable | Used in partial risk score (y/n) | | | Descriptive statistics | | |
|---|---|---|---|---|---|---|
| | OPOSSUM | SAPS II | NHFS | Median[d] | IQR[d] | Missing(%) |
| Age | y | y | y | 84.7 | 8.60 | 0.39 |
| Length of stay (days) | n | n | n | 8.10 | 8.60 | 0.00 |
| Charlson index (record-derived) | n | n | n | 0.89 | 1.58 | 0.00 |
| Number of previous admissions the last two years | n | n | n | 0.74 | 1.26 | 0.00 |
| Temperature (ºC) | n | y | n | 36.9 | 0.80 | 25 |
| Systolic blood pressure (mmHg) | y | y | n | 155 | 40.1 | 5.6 |
| Heart rate | y | y | n | 79.5 | 18.8 | 5.3 |
| Glasgow coma scale | n | n | n | 14.9 | 0.20 | 72 |
| Haemoglobin (g/100ml) | y | n | y | 12.5 | 2.10 | 1.7 |
| White blood cell count ($10^9$/l) | y | y[a] | n | 10.6 | 5.10 | 7.1 |
| Creatinine (µmol/l) | y | y | n | 76.4 | 35.9 | 3.0 |
| Sodium (mmol/l) | y | y | n | 140 | 3.80 | 1.2 |
| Potassium (mmol/l) | y | y | n | 84.7 | 8.60 | 0.39 |
| | | | | Not present(%) | Present(%) | Missing(%) |
| Female gender | n | n | y | 26 | 73 | 0.39 |
| Congestive heart failure | y | n | n | 89 | 11 | 0.0 |
| Comorbidities | n | n | y[c] | 38 | 62 | 0.0 |
| Lung disease | y | n | n | 88 | 12 | 0.0 |
| Dementia/delirium | n | n | y[b] | 78 | 22 | 0.0 |
| Pathological ECG | y | n | n | 21 | 39 | 40 |
| Metastatic cancer | y | y | n | 97 | 2.6 | 0.0 |
| Cancer | n | n | y | 87 | 13 | 0.0 |

| | OPOSSUM | SAPS II | NHFS | Not present(%) | Present(%) | Missing(%) |
|---|---|---|---|---|---|---|
| **HIV/AIDS** | n | y | n | 100 | 0.0 | 0.0 |
| **Emergency surgical** | n | y | n | 0.0 | 100 | 0.0 |
| **Admitted from long term care institution** | n | n | y | 89 | 11 | 0.0 |

[a]White blood cell count used as proxy for total lymphocyte count; [b]Dementia/delirium used as proxy for Mini-mental test (MMS) score; [c]Cardiovascular, respiratory or renal disease; IQR, Interquartile range. [d]Note that as sample stratification is accounted for when computing median and IQR, the values are not necessarily possible sample values
