# Supplementary material for: Variation between Hospitals with Regard to Diagnostic Practice, Coding Accuracy, and Case-Mix. A Retrospective Validation Study of Administrative Data versus Medical Records for Estimating 30-Day Mortality after Hip Fracture
Source: PLoS One. 2016 May 20;11(5):e0156075. doi: 10.1371/journal.pone.0156075 (PMC4874695; doi:10.1371/journal.pone.0156075)
Supplement: S3 Text — (PDF) [file pone.0156075.s004.pdf]

S3 Text. Comorbidity

Table A. Comparison of Charlson scores from medical records and from PAS

Source	Mean score	Percent score > 2
**Medical records**	1.4	17
**PAS**	0.89	11

Estimated using stratum weights. N=1043

Table B. Percentages of cases with individual Charlson comorbidities from medical records vs PAS

Comorbidity	Source	
	**Medical records**	**PAS**
Congestive heart failure	11	11
Dementia	22	9.6
Chronic pulmonary disease	12	7.8
Rheumatic disease	5.0	2.8
Mild liver disease	0.15	0.11
Diabetes with chronic complications	3.1	1.7
Hemiplegia/paraplegia	1.9	0.24
Renal disease	4.0	3.9
Malignancy	13	6.8
Moderate or severe liver disease	0.0	0.07
Metastatic solid tumor	2.6	2.9
AIDS/HIV	0.0	0.0

Estimated using stratum weights. N=1043
