# Supplementary material for: Variation between Hospitals with Regard to Diagnostic Practice, Coding Accuracy, and Case-Mix. A Retrospective Validation Study of Administrative Data versus Medical Records for Estimating 30-Day Mortality after Hip Fracture
Source: PLoS One. 2016 May 20;11(5):e0156075. doi: 10.1371/journal.pone.0156075 (PMC4874695; doi:10.1371/journal.pone.0156075)
Supplement: S4 Text — (PDF) [file pone.0156075.s005.pdf]

S4 Text. Double abstraction sub study

The classification of diagnosis, based on clinical signs and imaging evidence, of the two abstracters were 45/50 vs. 47/50 for definite hip fracture and 47/50 vs. 48/50 for probable hip fracture. The differences between abstracters were not significant ($p>0.5$). The relative mean differences for numerical (physiological and laboratory) variables had a median of 0.06% and a range of 0% - 6.1% over questionnaire items. The large value (6.1%) was due to a single clerical error.
